# Health-Related Social Needs Are Associated With Worse Physical Function, Pain, and Mobility in Hip and Knee Osteoarthritis Patients at Presentation

**DOI:** 10.1016/j.artd.2025.101871

**Published:** 2026-03-30

**Authors:** Adam D. Winter, Camila L. Hayashi, Luis A. Túa-Vargas, Nicholas Chau, David H. Gibson, Lee E. Rubin, Ilda B. Molloy, Daniel H. Wiznia

**Affiliations:** a Department of Orthopaedics and Rehabilitation, Yale School of Medicine, New Haven, CT, USA; b Department of Orthopaedics, Ponce Health Sciences University, Ponce, PR, USA

**Keywords:** Social determinants, Osteoarthritis, Arthroplasty, Patient-reported outcome measures

## Abstract

**Background:**

Health-related social needs (HRSNs) are mandatory reporting measures per the Centers for Medicare & Medicaid Services. We sought to assess the prevalence of HRSNs and associations with clinical presentation among preoperative hip and knee osteoarthritis patients.

**Methods:**

This prospective single-institution cross-sectional study assessed HRSNs (living situation, food, transportation, utilities, safety, financial strain, substance use, and mental health); Patient-Reported Outcomes Measurement Information System (PROMIS) Physical Function, Mobility, Pain Interference scores; and demographics (sex, race, education, income, household size, marital status) via in-person interviews in an academic arthroplasty practice. Kellgren-Lawrence Osteoarthritis Score, Charlson Comorbidity Index, and insurance were also determined. Odds of HRSNs by demographics were analyzed using logistic regression and relationship between HRSNs and PROMIS scores was determined by weighted least square linear regression.

**Results:**

The study included 253 patients. Prevalences of each HRSN were 15% for living situation, 13% for food insecurity, 11% for transportation, 4% for utilities, 0.4% for safety, 58% for mental health, 31% for substance use, and 27% for financial strain. Black patients and patients on Medicaid had higher HRSN prevalences. PROMIS scores were significantly worse in patients with HRSNs, particularly for living situation, financial strain, and mental health domains (*P* < .05 for Physical Function, Mobility, and Pain Interference scores in all these domains).

**Conclusions:**

HRSNs are significant among hip and knee osteoarthritis patients and disproportionately high for Black and Medicaid patients. Patients with HRSNs presented with significantly worse PROMIS scores across Physical Function, Mobility, and Pain Interference domains, highlighting a healthcare disparity in this population.

## Introduction

In the effort to improve healthcare outcomes for patients, increasing attention has been placed on health-related social needs (HRSNs) [1,2]. The Center for Medicare & Medicaid Services (CMS) defines 5 core domains of HRSNs (living situation, food insecurity, transportation, utilities, and safety) and 8 supplemental domains (mental health, substance use, financial strain, employment, education, physical activity, substance use, family and community support, and disability) [3]. Addressing these disparities is central to achieving CMS's mission of advancing health equity and improving the quality of care for underserved populations.

Various studies have demonstrated the relationship between HRSNs and hip and knee osteoarthritis (OA). Factors such as living condition, food insecurity, limited transportation access, lower socioeconomic status, and lower educational attainment have been linked to poor knee function, worse self-reported health outcomes, higher incidence rates of both radiographic and symptomatic OA, and increased likelihood of severe joint pain [[4], [5], [6], [7]]. Across these studies, it is increasingly evident that HRSNs impact the progression and overall patient experience of OA.

Multiple studies have also shown that HRSNs and OA are disproportionately associated with race and insurance type. Non-White adults, compared to White adults, experience higher prevalence rates of food insecurity, utility challenges, financial strain, and housing instability [8]. Black patients also experience a greater prevalence of lower extremity OA and severe radiographic knee OA, greater knee disability and pain, as well as worse hip OA severity compared to White patients [5,[9], [10], [11], [12]]. Patients with Medicaid insurance report significantly worse Physical Function scores prior to total joint arthroplasty (TJA) and to need to travel farther distances for healthcare access compared to those with private insurance [13,14].

Few publications screen for HRSNs and demographic information, measure their prevalence and correlation, and analyze which factors may be impacting patients with OA and their path to TJA. Identifying specific HRSNs associated with patient-reported outcome measures (PROMs), such as mobility and pain, as well as radiographic progression of OA, could help providers better support their patients in addressing these needs before undergoing TJA. This study aims to 1) collect data for 5 core HRSNs, 3 supplementary HRSNs, comorbidities, and demographics in preoperative OA patients; 2) examine relationships between demographic factors like race and insurance with HRSNs; and 3) correlate HRSNs and demographic data with radiographic OA scoring, comorbidities, and patient-reported metrics for pain, mobility, and physical function using Patient-Reported Outcomes Measurement Information System (PROMIS) testing. We hypothesize that Black patients and patients with Medicaid insurance will have higher prevalences of HRSNs and that patients with HRSNs will have more comorbidities, worse radiographic OA, and worse PROMIS scores (higher Pain Interference, lower Mobility, and lower Physical Function T-scores).

## Material and methods

### Data collection

This was a prospective single-institution cross-sectional study conducted in arthroplasty clinics at an academic tertiary center from May 2024 to August 2024. Data were gathered across 4 clinic sites: the main campus located in an urban setting as well as nearby suburban clinics in the same hospital system. Our institutional review board approved this study.

Eligibility criteria included all patients over 18 years old receiving care for hip or knee OA with one of the participating surgeons; exclusion criteria included patients who did not speak English and patients in clinic for a postoperative visit.

The HRSN Screening Tool, developed by CMS, was used to assess HRSNs among patients attending the clinics of 5 participating arthroplasty surgeons at an academic tertiary center [3]. This tool was designed to standardize the assessment of HRSNs and supports the agency's mandate to screen for and report HRSN information. All 5 of the core HRSN domains (living situation, food insecurity, transportation, utilities, and safety) were included in this study along with 3 of the supplemental HRSN domains (mental health, substance use, and financial strain) [3]. Definitions for a positive result using the HRSN Screening Tool were based on CMS criteria where positivity indicates the presence of a social need in the given domain (Appendix 1) [3]. Demographic information, including sex, race, annual household income, education level, number of cohabitants, and marital status, was also collected from the HRSN screener.

Eight patients declined to report income, one declined to answer cohabitants, and one declined to answer marital status. Kellgren-Lawrence (KL) grades could not be obtained for one patient because imaging was not in the electronic medical record (EMR). Charlson Comorbidity Index (CCI) was not calculated for 80 patients due to missing data in the EMR. Patients with missing values were excluded from any analysis using the missing variables.

The PROMIS Physical Function, Mobility, and Pain Interference computer adaptive tests were administered to assess the impact of OA on patients at the time of presentation [[15], [16], [17]]. PROMIS T-scores and associated standard error was determined for each patient. Higher T-scores represent better physical function, better mobility, and greater pain interference, respectively. Additional data were extracted from the EMR: insurance status, Kellgren-Lawrence Osteoarthritis Score (KL score—a common method of radiographically classifying OA severity), and CCI [[18], [19], [20]]. The patient's primary insurance was recorded if the patient was covered by more than one plan. Commercial insurance was defined as privately administered managed care plans, while Medicare included both traditional Medicare and Medicare Advantage plans.

Both the HRSN Screening Tool and PROMIS tests were administered by direct in-person patient interviews in the clinic setting. Tablets were used to conduct the survey and record patient answers. The study data were collected and managed using REDCap electronic data capture tools [21,22].

### Referral to resources

Patients who screened positive for any of the HRSN domains were referred to Community-Based Organizations (CBOs) to address their specific needs. Our institution used the UniteUs database (https://uniteus.com/) which is a social care software that aggregates CBOs and sorts them by specific services provided as well as distance to the patient's address [23]. Patients who requested resources were sent a list via text or email containing the contact information for relevant CBOs that they could use to reach out for assistance.

### Statistical analysis

Statistical analysis was conducted using Microsoft Excel and IBM SPSS Statistics (version 29). Independent sample *t*-tests were used to compare group means. Chi-squared test for independence or Fisher's exact test was used to compare distributions of categorical data. Mann-Whitney U test was used to compare group differences for ordinal variables when the assumption of normality was not met. A logistic regression was used to assess odds of HRSN positivity based on categorical variables (race and insurance status). A weighted least squares linear regression was used to analyze the difference in PROMIS score by HRSN status in each domain. The weights in the weighted least squares model were determined based on the standard error for each observed PROMIS T-score that is outputted from the computer adaptive test. We used a variance-weighted model, where each weight was calculated as the inverse of the variance, to give more weight to observed T-scores with higher precision and less weight to those with less precision. All tests were considered significant at 0.05.

## Results

A total of 253 patients who presented to the TJA clinic with OA completed the survey. The cohort was predominantly female (53%, 135) and White (74%, 187), with Black or African American patients comprising 21% (54) and patients of Hispanic, Latino/a, or Spanish origin making up 6% (15). The median annual household income fell within the $50,000-$75,000 bracket (interquartile range: $25,000-$35,000 to >$75,000). This is lower than the 2023 U.S. Census reported national median household income of $80,610 [24]. Up to 71% (180) of our population had completed at a minimum of some college, associate's, or vocational training. Thirty-one percent (78) of patients lived alone. Regarding insurance, 51% (127) had Medicare or Medicare Advantage, 21% (53) were covered by Medicaid, and 29% (73) had private insurance. This differs from the national distribution of Medicare (18.9%), Medicaid (18.9%), and private insurance (53.7%); our population had higher proportions of Medicare and Medicaid patients and fewer with private insurance [24]. The majority of patients had advanced OA, with 24% (60) classified as KL Grade 3 and 68% (172) as Grade 4 (Table 1).

**Table 1:** Demographics and prevalence of HRSN among total joint arthroplasty patients.

Variable	Value (N = 253)
Sex	
Female	135 (53%)
Male	118 (47%)
Race^a^	
White	187 (74%)
Black or African American	54 (21%)
Hispanic, Latino/a, or Spanish origin	15 (6%)
Other^b^	13 (5%)
Annual household income	
<$10,000	15 (6%)
$10,000-$15,000	21 (8%)
$15,000-$20,000	7 (3%)
$20,000-$25,000	11 (4%)
$25,000-$35,000	21 (8%)
$35,000-$50,000	25 (10%)
$50,000-$75,000	31 (12%)
>$75,000	114 (45%)
Education	
Elementary	6 (2%)
Some high school	9 (4%)
High school graduate or GED	58 (23%)
Some college, associate’s degree, or vocational school	60 (24%)
College graduate	120 (47%)
Number of cohabitants	
Live alone	78 (31%)
Live with one other person	105 (42%)
Live with 2 or more people	69 (27%)
Marital status	
Married	111 (44%)
Divorced	48 (19%)
Never married	47 (19%)
Widowed	24 (9%)
Living with a partner	18 (7%)
Separated	4 (2%)
Insurance	
Commercial	73 (29%)
Medicare	127 (50%)
Medicaid	53 (21%)
Health-related social needs	
Mental health	147 (58%)
Substance use	79 (31%)
Financial strain	69 (27%)
Living situation^c^	39 (15%)
Food insecurity^c^	33 (13%)
Transportation^c^	27 (11%)
Utilities^c^	10 (4%)
Safety^c^	1 (0.4%)
Number of HRSNs present	
0 categories	72 (29%)
1 categories	77 (30%)
2 categories	43 (17%)
3 categories	29 (12%)
4 categories	14 (6%)
5 categories	9 (4%)
6 categories	9 (4%)
Kellgren-Lawrence Osteoarthritis Score	
Grade 0-1	4 (2%)
Grade 2	16 (6%)
Grade 3	60 (24%)
Grade 4	172 (68%)
Charlson Comorbidity Index	Median = 1.00 (IQR: 0.00-3.00)

IQR, interquartile range; GED, general education development.

Values listed as n (%).

a Individuals selecting more than one race were counted in multiple categories.

b Includes American Indian/Alaska Native, Asian, Native Hawaiian/other Pacific Islander, and other.

c Indicates CMS core domain for HRSN.

Of the core HRSN domains, the highest positivity rates were for living situation = 15% (39), food insecurity = 13% (33), and transportation = 11% (27), where positivity is defined as having a social need in that domain. Among supplemental domains, positivity rates were mental health = 58% (147), substance use = 31% (79), and financial strain = 27% (69). The majority of patients screened positive for at least one HRSN domain (72%, 181), and 13% (32) were positive for 4 or more HRSNs (Table 1). There was no significant difference in the median CCI or KL score for patients positive vs negative in each HRSN category.

Black patients and patients on Medicaid were found to have a higher burden of HRSNs (Table 2). With logistic regression analysis, Black patients had higher odds of a HRSN in the living situation (odds ratio [OR]: 2.49; 95% confidence interval [CI]: 1.18-5.28; *P* = .017), food (OR: 3.84; 95% CI: 1.76-8.36; *P* = .001), transportation (OR: 2.75; 95% CI: 1.18-6.38; *P* = .019), and financial strain (OR: 2.16; 95% CI: 1.14-4.10; *P* = .018) domains, compared to White patients. Medicaid patients were found to have higher odds of a HRSN in the living situation (OR: 3.63; 95% CI: 1.28-10.30; *P* = .015), food (OR: 43.64; 95% CI: 5.62-339.03; *P* < .001), transportation (OR: 7.58; 95% CI: 2.04-28.22; *P* = .003), financial strain (OR: 7.62; 95% CI: 3.36-17.24; *P* < .001), and substance use (OR: 3.53; 95% CI: 1.68-7.43; *P* = .001) domains.

**Table 2:** Prevalence of each HRSN by race and insurance status.

Result of HRSN screen by demographic group	Negative	Positive	*P* value	OR	95% CI for OR	
	n (%)	n (%)			Upper	Lower
Living situation						
White	163 (87.20%)	24 (12.80%)	-	Ref	-	-
Black	40 (74.10%)	14 (25.90%)	**0.017**	2.49	1.18	5.28
Hispanic	14 (93.30%)	1 (6.70%)	0.342	0.37	0.05	2.91
Other race	11 (84.60%)	2 (15.40%)	0.683	1.39	0.29	6.75
Commercial insurance	67 (91.80%)	6 (8.20%)	-	Ref	-	-
Medicaid	40 (75.50%)	13 (24.50%)	**0.015**	3.63	1.28	10.3
Medicare	107 (84.30%)	20 (15.70%)	0.134	2.09	0.8	5.46
Food insecurity						
White	170 (90.90%)	17 (9.10%)	-	Ref	-	-
Black	39 (72.20%)	15 (27.80%)	**0.001**	3.84	1.76	8.36
Hispanic	12 (80.00%)	3 (20.00%)	0.372	1.87	0.47	7.42
Other race	12 (92.30%)	1 (7.70%)	0.806	0.77	0.09	6.36
Commercial insurance	72 (98.60%)	1 (1.40%)	-	Ref	-	-
Medicaid	33 (62.30%)	20 (37.70%)	**<0.001**	43.64	5.62	339.03
Medicare	115 (90.60%)	12 (9.40%)	0.055	7.51	0.96	59.01
Transportation						
White	171 (91.40%)	16 (8.60%)	-	Ref	-	-
Black	43 (79.60%)	11 (20.40%)	**0.019**	2.75	1.18	6.38
Hispanic	15 (100.00%)	0 (0.00%)	0.998	0	0	-
Other race	13 (100.00%)	0 (0.00%)	0.999	0	0	-
Commercial insurance	70 (95.90%)	3 (4.10%)	-	Ref	-	-
Medicaid	40 (75.50%)	13 (24.50%)	**0.003**	7.58	2.04	28.22
Medicare	116 (91.30%)	11 (8.70%)	0.235	2.21	0.6	8.21
Utilities						
White	182 (97.30%)	5 (2.70%)	-	Ref	-	-
Black	50 (92.60%)	4 (7.40%)	0.122	2.91	0.75	11.27
Hispanic	15 (100.00%)	0 (0.00%)	0.999	0	0	-
Other race	12 (92.30%)	1 (7.70%)	0.282	3.42	0.36	32.12
Commercial insurance	71 (97.30%)	2 (2.70%)	-	Ref	-	-
Medicaid	50 (94.30%)	3 (5.70%)	0.417	2.13	0.34	13.22
Medicare	122 (96.10%)	5 (3.90%)	0.659	1.45	0.28	7.7
Safety						
White	186 (99.50%)	1 (0.50%)	-	Ref	-	-
Black	54 (100.00%)	0 (0.00%)	0.998	0	0	-
Hispanic	15 (100.00%)	0 (0.00%)	0.999	0	0	-
Other race	13 (100.00%)	0 (0.00%)	0.999	0	0	-
Commercial insurance	73 (100.00%)	0 (0.00%)	-	Ref	-	-
Medicaid	52 (98.10%)	1 (1.90%)	0.997	>999.99	0	-
Medicare	127 (100.00%)	0 (0.00%)	1	1	0	-
Mental health						
White	79 (42.20%)	108 (57.80%)	-	Ref	-	-
Black	22 (40.70%)	32 (59.30%)	0.821	1.07	0.58	1.99
Hispanic	6 (40.00%)	9 (60.00%)	0.897	1.07	0.37	3.13
Other race	5 (38.50%)	8 (61.50%)	0.787	1.17	0.37	3.74
Commercial insurance	27 (37.00%)	46 (63.00%)	-	Ref	-	-
Medicaid	12 (22.60%)	41 (77.40%)	0.088	2.01	0.9	4.46
Medicare	67 (52.80%)	60 (47.20%)	**0.032**	.53	0.29	0.95
Substance use						
White	134 (71.70%)	53 (28.30%)	-	Ref	-	-
Black	31 (57.40%)	23 (42.60%)	0.052	1.86	1	3.48
Hispanic	10 (66.70%)	5 (33.30%)	0.81	1.15	0.37	3.53
Other race	10 (76.90%)	3 (23.10%)	0.661	0.74	0.2	2.82
Commercial insurance	51 (69.90%)	22 (30.10%)	-	Ref	-	-
Medicaid	21 (39.60%)	32 (60.40%)	**0.001**	3.53	1.68	7.43
Medicare	102 (80.30%)	25 (19.70%)	0.095	0.57	0.29	1.1
Financial Strain						
White	141 (75.40%)	46 (24.60%)	-	Ref	-	-
Black	32 (59.30%)	22 (40.70%)	**0.018**	2.16	1.14	4.10
Hispanic	9 (60.00%)	6 (40.00%)	0.214	2.01	0.67	6.04
Other race	11 (84.60%)	2 (15.40%)	0.415	0.52	0.11	2.49
Commercial insurance	60 (82.20%)	13 (17.80%)	-	Ref	-	-
Medicaid	20 (37.70%)	33 (62.30%)	**<0.001**	7.62	3.36	17.24
Medicare	104 (81.90%)	23 (18.10%)	0.957	1.02	0.48	2.16

OR, odds ratio; CI, confidence interval.

Logistic regression predicts odds of screening positive in each HRSN domain based on the demographic variable.

Significant *P* values are bolded.

Patients with HRSNs demonstrated significantly worse PROMIS T-scores across Physical Function, Mobility, and Pain Interference domains (Table 3). PROMIS-Physical Function scores were worse in patients with living situation (*P* = .010), transportation (*P* = .026), mental health (*P* = .023), and financial strain (*P* = .017) needs. PROMIS-Mobility scores were worse in patients with living situation (*P* = .003), mental health (*P* < .001), and financial strain (*P* = .002) needs. PROMIS-Pain Interference scores were worse in patients with living situation (*P* = .003), food insecurity (*P* < .001), transportation (*P* = .011), mental health (*P* < .001), substance use (*P* < .001), and financial strain (*P* < .001) needs. Higher T-scores indicate better physical function and mobility but more pain interference. Significant P values bolded for visibility.

**Table 3:** PROMIS scores by HRSN status.

HRSN	Physical function			Mobility			Pain interference		
	HRSN (−) cohort PROMIS T-score weighted estimate ± std. error	Estimated difference in weighted T-score for HRSN (+)	*P* value	HRSN (−) cohort PROMIS T-score weighted estimate ± std. error	Estimated difference in weighted T-score for HRSN (+)	*P* value	HRSN (−) cohort PROMIS T-score weighted estimate ± std. error	Estimated difference in weighted T-score for HRSN (+)	*P* value
Living situation	39.7 ± 0.5	−3.1 ± 1.2	**.010**	37.4 ± 0.5	−3.6 ± 1.2	**.003**	60.7 ± 0.5	3.4 ± 1.1	**.003**
Food insecurity	39.5 ± 0.5	−1.8 ± 1.3	.148	37.2 ± 0.5	−2.4 ± 1.3	.061	60.7 ± 0.4	4.4 ± 1.3	**<.001**
Transportation	39.6 ± 0.5	−3.2 ± 1.4	**.026**	37.2 ± 0.5	−2.8 ± 1.4	.050	60.8 ± 0.4	3.6 ± 1.4	**.011**
Utilities	39.3 ± 0.4	−1.0 ± 2.2	.656	37.0 ± 0.4	−1.5 ± 2.2	.510	61.1 ± 0.4	2.4 ± 2.3	.288
Safety	39.3 ± 0.4	−6.6 ± 6.5	.315	36.9 ± 0.4	−4.5 ± 6.4	.482	61.2 ± 0.4	−1.3 ± 5.9	.823
Mental health	40.4 ± 0.7	−2.0 ± 0.9	**.023**	38.7 ± 0.6	−3.1 ± 0.9	**<.001**	58.8 ± 0.6	4.0 ± 0.8	**<.001**
Substance use	39.2 ± 0.5	0.2 ± 0.9	.868	37.3 ± 0.5	−1.1 ± 0.9	.243	60.2 ± 0.5	3.1 ± 0.9	**<.001**
Financial strain	39.9 ± 0.5	−2.3 ± 1.0	**.017**	37.7 ± 0.5	−3.0 ± 1.0	**.002**	60.1 ± 0.5	4.2 ± 0.9	**<.001**

(+) indicates the presence of a HRSN in the domain listed. Variance-weighted PROMIS T-score estimates listed, where higher score represents better physical function, better mobility, and more interference from pain, respectively. P values and standard error from coefficients of weighted least squares regression.

Significant *P* values are bolded.

## Discussion

We used the CMS HRSN screening tool to survey hip and knee OA patients in an academic tertiary healthcare setting and found that 1) the prevalences of HRSNs—especially food insecurity, transportation issues, housing insecurity, and mental health—were higher than have been previously reported; 2) Black patients and patients on Medicaid have a disproportionately higher prevalence of HRSNs; and 3) several HRSN domains—including living situation, financial strain, and mental health—are significantly associated with worse PROMs in pain, physical function, and mobility at presentation.

### Prevalence of HRSNs

We screened for the 5 core HRSN domains (living situation, food insecurity, transportation, utilities, and safety) and 3 supplemental domains (mental health, substance use, and financial strain). We found that the prevalences of food insecurity, transportation issues, and housing insecurity among hip and knee OA patients were 13%, 11%, and 15%, respectively. Notably, 71% of patients in our study reported at least one HRSN. In a study by Norris et al. in which they surveyed 250 primary TJA patients in a tertiary-referral outpatient clinic, just 17.6% reported at least one HRSN [25]. Their analysis found that only 1.2% of patients reported food insecurity, 3.6% reported transportation needs, and 2.4% reported housing instability [25]. Differences in our HRSN results compared to Norris et al. may be due to the geographic area of study (urban vs rural) or differences in survey administration (composition of questions and the mode of delivery). Nationally, the prevalences of food insecurity, transportation barriers to healthcare, and housing insecurity are estimated to be 13.5%, 10%-51%, and 10%-15%, respectively [[26], [27], [28]]. Our population demographics are more similar to existing data for arthroplasty patients in urban tertiary care settings. Ruff et al. found that at their urban academic medical center, 25.8%, 19.3%, and 54.9% of total hip arthroplasty (THA) patients had commercial insurance, Medicaid, and Medicare, respectively [29]. The comparison of our HRSN prevalence data with national averages and of our demographic data with patient populations seeking arthroplasty care in urban tertiary care settings suggest that the prevalence of HRSNs among pre-TJA patients may be higher than previously recognized. These data and future collection of HRSNs can help clinics prioritize community resources based on patient needs and provide insight to inform a biopsychosocial model of care.

Regarding mental health HRSNs, 58% of patients screened positive for a mental health concern, a notably higher prevalence compared to published data. Previous studies report prevalences ranging from 20% to 32% for symptoms of anxiety or depression among preoperative TJA patients [30,31]. These studies leveraged other screening methods to capture mental health HRSNs that may have less sensitivity compared to screening tools like the Patient Health Questionnaire-2, which is integrated into the CMS HRSN survey [32]. We suspect that the broad screening approach of the CMS tool used in this study contributed to the higher prevalence of mental health HRSNs and that this high prevalence of mental health HRSNs is not necessarily a unique characteristic of our population.

### HRSNs and demographic information

When examining race and HRSNs, we found that patients who identified as Black or African American were disproportionately impacted by HRSNs compared to White patients. Black patients had significantly higher rates of positive screenings for housing insecurity, food insecurity, transportation, and financial strain, consistent with prior research [7,25]. Similarly, when examining insurance type and HRSNs, we found that patients who were insured with Medicaid had higher prevalences of HRSNs compared to patients with commercial insurance. Medicaid patients were more likely to screen positive for housing insecurity, transportation, financial strain, and substance use, highlighting a greater level of need among Medicaid patients.

### HRSNs and patient-reported outcome measures

Significant associations were observed between several HRSNs and pre-TJA PROMs. Among core HRSNs, living situation impacted all 3 PROMIS measures, while transportation was associated with worse Physical Function and Pain Interference scores, and food insecurity correlated with worse Pain Interference scores. Our findings align with similar trends showing that HRSNs negatively affect patient outcomes. Living situation has been linked to worse self-reported health, severe OA pain, and lower Knee Injury and Osteoarthritis Outcome Score for Joint Replacement scores [4,7,33]. Lack of reliable transportation contributes to delays in care and missed appointments, while food insecurity, although not directly studied in OA, is associated with malnutrition, which worsens TJA outcomes [27,34,35].

Of the supplemental HRSNs, we found mental health and financial strain had significant negative associations to all 3 PROMIS scores. Previous work has shown that preoperative anxiety and depressive symptoms predicted significantly lower improvements in acts of daily living and quality of life measurements via Hip dysfunction and Osteoarthritis Outcome Score and Knee Injury and Osteoarthritis Outcome Score for Joint Replacement scoring after TJA, pointing to the impact of mental health on TJA recovery and patient-reported metrics [30]. We found that patients reporting financial strain had significant negative associations with all 3 PROMIS measures. This result is consistent with literature demonstrating that low income has been associated with higher KL grades, poorer hip and knee functional scores, higher OA pain, and increased limitations on acts of daily living [6,36,37].

### Limitations

This study has several limitations. This was a cross-sectional study, so any causal relationships between HRSNs and severity of OA presentation cannot be determined. Due to the single-institution design, there is an inherent geographic selection bias that may limit generalizability of data to other settings. Additionally, due to the nature of in-person data collection in a clinic setting, it is unknown how many potentially eligible patients were unable to be surveyed. However, every attempt was made to survey patients when eligible. For some patients, incomplete EMR data limited the inclusion of patients with missing data in an analysis. In particular, analysis determining significant differences in CCI scores for patients with or without HRSNs excluded 80 patients (31.6%) for whom CCI scores were missing despite full HRSN data. This potentially masked a true difference in CCI between the groups due to selection bias or lack of statistical power. Finally, the validity of our HRSN data is based on what patients reported; survey data like income or presence of an HRSN were not verified by external sources.

Given the complexity of TJA preparation and recovery, including appointments, discharge coordination, and wound care, addressing HRSNs is critical. Optimizing patients for surgery requires meeting benchmarks for body mass index, nutritional markers, tobacco cessation, and medication adjustments [[38], [39], [40]]. Based on our results, we recommend that preoperative optimization programs focus on addressing housing, transportation, food, and mental health, as these needs have a high prevalence and align with optimization goals and support improved outcomes. Referrals to housing programs, transportation services, food assistance, and mental health resources could mitigate these barriers and enhance pre- and post-surgical care for TJA patients.

## Conclusions

In a large, prospectively collected, cross-sectional sample of hip and knee OA patients, the present study identified a high prevalence of the CMS-defined HRSNs across many domains including food insecurity, transportation issues, housing insecurity, and mental health. Certain subgroups, including Black and Medicaid patients, were found to have a disproportionately high burden of HRSNs. Furthermore, the presence of HRSNs was associated with worse patient-reported physical function, mobility, and pain at presentation. To our knowledge, this is the first study to quantify the association between the CMS-defined HRSNs and a more severe baseline clinical presentation in hip and knee OA patients. These findings can serve as an evidence-based benchmark for future efforts to track HRSNs, accurately measure the clinical impact, and assess progress toward health equity in the arthroplasty patient population.

## Conflicts of interest

L.E. Rubin is a paid consultant for 10.13039/100008127DePuy Synthes, Innovative Medical Products, and 10.13039/100004686Thompson Surgical Instruments; receives royalties, financial, or material support from SLACK, Taylor & Francis, 10.13039/100019748Johns Hopkins University Press, and Wolters Kluwer; is a member of Medical publications editorial/governing board of *Journal of Arthroplasty* and *Arthroplasty Today*. I.B. Molloy is a paid consultant for Smith & Nephew; all other authors declare no potential conflicts of interest.

For full disclosure statements refer to https://doi.org/10.1016/j.artd.2025.101871

## CRediT authorship contribution statement

**Adam D. Winter:** Writing – review & editing, Writing – original draft, Visualization, Validation, Supervision, Software, Resources, Project administration, Methodology, Investigation, Formal analysis, Data curation, Conceptualization. **Camila L. Hayashi:** Writing – review & editing, Writing – original draft, Visualization, Validation, Supervision, Software, Resources, Project administration, Methodology, Investigation, Formal analysis, Data curation, Conceptualization. **Luis A. Túa-Vargas:** Writing – review & editing, Writing – original draft, Visualization, Validation, Supervision, Software, Resources, Project administration, Methodology, Investigation, Formal analysis, Data curation, Conceptualization. **Nicholas Chau:** Writing – review & editing, Writing – original draft, Visualization, Validation, Supervision, Software, Resources, Project administration, Methodology, Investigation, Formal analysis, Data curation, Conceptualization. **David H. Gibson:** Writing – review & editing, Writing – original draft, Visualization, Validation, Supervision, Resources, Project administration, Methodology, Investigation, Data curation, Conceptualization. **Lee E. Rubin:** Writing – review & editing, Writing – original draft, Visualization, Validation, Supervision, Resources, Project administration, Methodology, Investigation, Data curation, Conceptualization. **Ilda B. Molloy:** Writing – review & editing, Writing – original draft, Visualization, Validation, Supervision, Resources, Project administration, Methodology, Investigation, Data curation, Conceptualization. **Daniel H. Wiznia:** Writing – review & editing, Writing – original draft, Visualization, Validation, Supervision, Resources, Project administration, Methodology, Investigation, Formal analysis, Data curation, Conceptualization.

## Appendix A Supplementary data

Conflict of Interest Statement for Chau

Conflict of Interest Statement for Túa-Vargas

Conflict of Interest Statement for Hayashi

Conflict of Interest Statement for Winter

Conflict of Interest Statement for Gibson

Conflict of Interest Statement for Molloy

Conflict of Interest Statement for Rubin

Conflict of Interest Statement for Wiznia
