# Supplementary material for: Health-Related Social Needs Are Associated With Worse Physical Function, Pain, and Mobility in Hip and Knee Osteoarthritis Patients at Presentation
Source: Arthroplast Today. 2026 Mar 30;37(Suppl):101871. doi: 10.1016/j.artd.2025.101871 (PMC13237586; doi:10.1016/j.artd.2025.101871)
Supplement: Conflict of Interest Statement for Túa-Vargas [file mmc2.pdf]

CONFLICT OF INTEREST STATEMENT

American Association of Hip and Knee Surgeons

(Adopted from the American Academy of Orthopaedic Surgeons disclosure statement)

The following form **must be filled out completely and submitted by each author (example, 6 authors, 6 forms). All items require a response. If there is no relevant disclosure for a given item, enter "*None*."**

Manuscript Title: ***Health-Related Social Needs are Associated with Worse Physical Function, Pain, and Mobility in Hip and Knee Osteoarthritis Patients at Presentation***

1. Royalties from a company or supplier (The following conflicts were disclosed)
   NONE

2. Speakers bureau/paid presentations for a company or supplier (The following conflicts were disclosed)

   NONE

3A. Paid employee for a company or supplier (The following conflicts were disclosed)

   NONE

3B. Paid consultant for a company or supplier (The following conflicts were disclosed)

   NONE

3C. Unpaid consultants for a company or supplier (The following conflicts were disclosed)

   NONE

4. Stock or stock options in a company or supplier (The following conflicts were disclosed)

   NONE

5. Research support from a company or supplier as a Principal Investigator (The following conflicts were disclosed)

   NONE

6. Other financial or material support from a company or supplier (The following conflicts were disclosed)

   NONE

7. Royalties, financial or material support from publishers (The following conflicts were disclosed)

   NONE

8. Medical/Orthopaedic publications editorial/governing board (The following conflicts were disclosed)

   NONE

9. Board member/committee appointments for a society (The following conflicts were disclosed)

   NONE

Each author must sign AND print or type his/her name, date and submit a separate form

In addition, one BLINDED Conflict of Interest form (no author names used) should be submitted per manuscript with all author disclosures.

**Luis A. Túa-Vargas**	[signature]	**01/25/2025**
Author Name (Print or Type)	Author Signature	Date
