# Supplementary material for: Health-Related Social Needs Are Associated With Worse Physical Function, Pain, and Mobility in Hip and Knee Osteoarthritis Patients at Presentation
Source: Arthroplast Today. 2026 Mar 30;37(Suppl):101871. doi: 10.1016/j.artd.2025.101871 (PMC13237586; doi:10.1016/j.artd.2025.101871)
Supplement: Conflict of Interest Statement for Rubin [file mmc7.pdf]

CONFLICT OF INTEREST STATEMENT

American Association of Hip and Knee Surgeons

(Adopted from the American Academy of Orthopaedic Surgeons disclosure statement)

The following form **must be filled out completely and submitted by each author (example, 6 authors, 6 forms). All items require a response. If there is no relevant disclosure for a given item, enter "*None.*"**

Manuscript Title: Health-Related Social Needs are Associated with Worse Physical Function, Pain, and Mobility in Hip and Knee Osteoarthritis Patients at Presentation

1. Royalties from a company or supplier (The following conflicts were disclosed)
NONE

2. Speakers bureau/paid presentations for a company or supplier (The following conflicts were disclosed)
NONE

3A. Paid employee for a company or supplier (The following conflicts were disclosed)
NONE

3B. Paid consultant for a company or supplier (The following conflicts were disclosed)
DePuy Synthes, Innovative Medical Products, Thompson Surgical Instruments

3C. Unpaid consultants for a company or supplier (The following conflicts were disclosed)
NONE

4. Stock or stock options in a company or supplier (The following conflicts were disclosed)
NONE

5. Research support from a company or supplier as a Principal Investigator (The following conflicts were disclosed)
NONE

6. Other financial or material support from a company or supplier (The following conflicts were disclosed)
NONE

7. Royalties, financial or material support from publishers (The following conflicts were disclosed)
SLACK, Taylor & Francis, Johns Hopkins University Press, Wolters Kluwer

8. Medical/Orthopaedic publications editorial/governing board (The following conflicts were disclosed)
Journal of Arthroplasty, Arthroplasty Today

9. Board member/committee appointments for a society (The following conflicts were disclosed)
NONE

Each author must sign AND print or type his/her name, date and submit a separate form

In addition, one BLINDED Conflict of Interest form (no author names used) should be submitted per manuscript with all author disclosures.

Lee Eric Rubin, MD	Lee E. Rubin, M.D.<br>Signed Electronically 1-20-2025 21:18 LER	January 20, 2025
Author Name (Print or Type)	Author Signature	Date
